# Direct Percutaneous Embolization of Peristomal Ileostomy Varices in an Emergency Setting

**DOI:** 10.1155/2018/6239183

**Published:** 2018-08-08

**Authors:** William Ryan, Farouk Dako, Gary Cohen, David Pryluck, Joseph Panaro, Emily Cuthbertson, Dmitry Niman

**Affiliations:** ^1^Lewis Katz School of Medicine at Temple University, USA; ^2^Temple University Hospital, USA

## Abstract

Patients with liver disease and portal hypertension who have had surgical formation of an abdominal stoma are at risk of developing peristomal varices. These varices have a predilection for bleeding. Ideally, portal decompression via TIPS procedure is performed, with or without direct embolization of the bleeding varix. When TIPS is not an appropriate option due to significant liver disease and hepatic encephalopathy there are other approaches to treat peristomal variceal hemorrhage. We report the embolization of such a varix via direct percutaneous puncture under ultrasound guidance when portal decompression was not an appropriate option.

## 1. Intro

Ectopic varices are defined as varices found outside of the esophageal or gastric mucosae [1]. Similar to esophageal and gastric varices, they are commonly related to portal hypertension and can cause clinically significant hemorrhage. Patients with portal hypertension who undergo surgical enterostomy formation have a tendency to form peristomal ectopic varices, and these varices are prone to bleeding [2, 3]. Mortality from these peristomal variceal bleeds is estimated to be 3-4% per episode; thus many of these patients undergo rapid intervention aimed at either embolization, portal venous decompression, or a combination of the two [1]. Because these cases are not especially common, comprising only about 5% of variceal bleeding, no consensus has been reached on optimal interventional approach [4]. A review of the literature illustrates that the percutaneous transhepatic approach, transjugular transhepatic approach, balloon-occluded retrograde transvenous occlusion technique, and direct varix puncture have all shown varying degrees of success in controlling peristomal variceal hemorrhage, with the latter being the least reported upon. We present a case of peristomal variceal hemorrhage that was successfully treated via direct percutaneous access of an engorged peristomal varix under ultrasound guidance and subsequent embolization with coils and Gelfoam to the feeding portal venous flow.

## 2. Case

A 59-year-old woman presented in July 2017 with extensive bleeding from her ileostomy site. Her history included locally advanced bladder cancer for which she had undergone pelvic exenteration and ileal conduit formation in November 2015. At that time, she had a known primary lung adenocarcinoma as well, but had no known liver metastases or other liver disease. Intravenous contrast-enhanced CT of the abdomen and pelvis performed in January 2016 raised the possibility of cirrhosis; however this was not biopsy-proven. In April 2016, she began to notice intermittent bleeding from her stoma which was initially thought to be mechanical tissue breakdown from the stomal flange. Concern for hepatic encephalopathy was raised when she had her first episode of confusion in December 2016. At that time CT of the abdomen and pelvis demonstrated strong radiographic suspicion for cirrhosis together with prominent vessels surrounding the urinary diversion site suspicious for portal hypertension. Despite not having a tissue biopsy, she was diagnosed clinically with cryptogenic cirrhosis in May 2017 during a hospitalization for fatigue, anasarca, and altered mental status. An upper endoscopy performed in June 2017 demonstrated portal hypertensive gastropathy but no esophageal varices.

**Figure 1:**
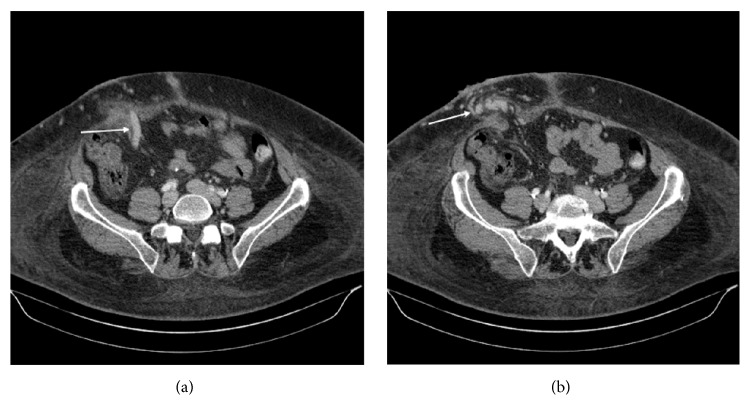
IV contrast-enhanced CT images of the abdomen and pelvic. (a) Large draining varix from the portal venous system (arrow). (b) Multiple varices within the right lower quadrant stoma (arrow).

Upon presentation to the Emergency Department in July 2017 she had significant hemorrhage from her stoma resulting in hemodynamic instability. She was anemic with a hemoglobin of 8.3 g/dL that improved to 9.4 g/dL after blood transfusion, but gradually fell to 8.2 g/dL by the time of the procedure. Her MELD score was retrospectively calculated to be 19 at presentation, with an INR of 1.5 and total bilirubin of 4.3 mg/dl. She was emergently taken to the interventional suite for embolization with or without portal venous decompression via portosystemic shunt formation. A review of intravenous contrast-enhanced CT imaging showed extensive venous varices around the stoma involving the abdominal wall with a large draining varix arising from the portal system, likely the inferior mesenteric vein [Figure 1]. Also visualized was a variceal connection to the right common femoral vein. The portal and mesenteric veins were noted to be patent. Multiple approaches were considered for this patient. The transjugular intrahepatic portosystemic shunt (TIPS) and transjugular transhepatic approach with portosystemic shunt creation offered the benefit of portal decompression; however, the patient's recurrent hepatic encephalopathy was felt to be a relative contraindication. Transjugular transhepatic approach without formation of a permanent portosystemic shunt was also considered, since it would eliminate the risk of progressive hepatic encephalopathy. Percutaneous transhepatic approach would also eliminate the risk of progressive hepatic encephalopathy but was believed to pose increased risks of hepatic injury and bleeding. Transsplenic venous access to the portal venous system was considered as a viable, albeit technically challenging, option. The superficial nature of the abdominal wall stomal varix presented a less challenging and seemingly more time-efficient approach for access and was chosen as the target.

**Figure 2:**
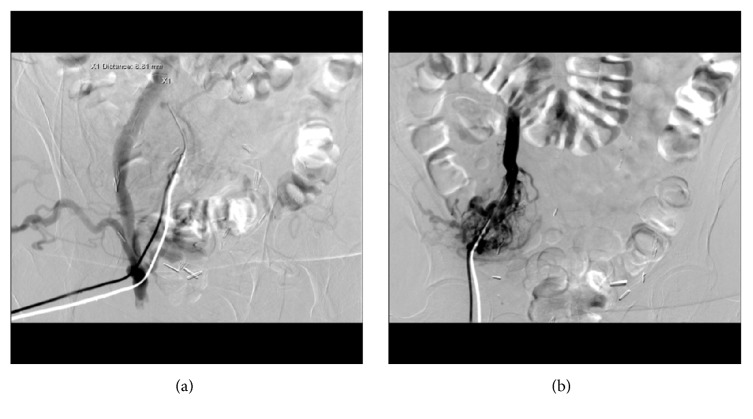
Fluoroscopic images of right lower quadrant. (a) Contrast injection of the peristomal varix and opacification of the right iliac vein. (b) Opacification of the peristomal varices and the right iliac vein.

**Figure 3:**
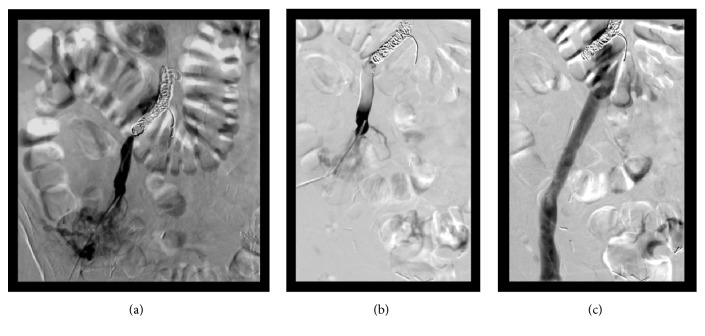
Fluoroscopic images of the right lower quadrant. (a) Venography after embolization with coils demonstrates hemostasis. (b) Subsequent venography after embolization with glue demonstrated sluggish flow in the portosystemic shunt with multiple filling defects within the visualized collaterals consistent with embolization. (c) Injection of the right femoral vein demonstrated flow from the right common femoral vein through the iliac system and IVC without opacification of the peristomal varices.

Using a micropuncture kit, the peristomal varix was directly accessed under ultrasound guidance and a micropuncture sheath was placed. Venography was performed and showed a large variceal collateral conglomerate around the stoma with variceal anastomosis with the right common femoral vein [Figure 2]. A wire was advanced and a 5F sheath was secured over the wire. A Kumpe catheter was introduced and advanced into the distal intra-abdominal aspect of the large draining varix. Catheter position was confirmed with repeat venography, and multiple coils were deployed [Figure 3]. This was followed by Gelfoam embolization. Postembolization venography showed sluggish flow in the draining varix with multiple filling defects within the visualized collaterals consistent with embolization [Figure 3]. The coils remained well-situated after placement and there was no evidence of migration. To ensure that there was no filling from the systemic venous system, the right superficial femoral vein was then accessed with a micropuncture kit and a femoral-iliac venogram and IVC venogram were both performed. These demonstrated brisk flow from the right common femoral vein through the iliac system and into the IVC. There was no filling of the stomal variceal collaterals visualized [Figure 3]. Hemostasis was thereby achieved, and the patient became hemodynamically stable shortly thereafter. At 6-month follow-up time no further imaging had been performed and the patient had not had any further episodes of hemorrhage from the ileostomy site

## 3. Discussion

Ectopic varices likely account for up to 5% of all variceal bleeding [4]. While the exact prevalence of peristomal variceal bleeding is unknown, one review indicated that of 169 ectopic variceal bleeds, 17% (26/169) of them were peristomal in nature [1]. An episode of peristomal variceal bleeding in the setting of portal hypertension carries an estimated mortality rate of 3-4% per episode, thus understanding different mechanisms of treatment is important [1].

The creation of an abdominal wall stoma on its own does not necessarily pose a significant risk of peristomal varix formation. In a review of 244 patients without prior liver disease it was found that only 1.5% of patients with ileal conduit formation went on to develop peristomal varices [5]. The problem ensues when liver disease and portal hypertension are present concurrently with an abdominal wall stoma. Estimates from 27-53% of patients with chronic liver disease and ileostomy/colostomy creation will go on to develop peristomal varices [2, 3]. Hemorrhage requiring blood transfusion may occur in up to 70% of these patients [3]. The specific etiology and severity of liver disease and reason for small bowel/colonic stomal formation may mediate the risk of varix development. For example, patients with primary sclerosing cholangitis (PSC) have an up to 90% risk of underlying ulcerative colitis (UC) [6]. The progressive hepatic fibrosis, driven by PSC, and the high likelihood of colonic resection with stoma formation, driven by UC, put these patients at a significant risk for developing peristomal varices [3].

When a peristomal varix begins to bleed, there are multiple approaches to achieve hemostasis, none of which have been deemed “first line”. Because portal hypertension is the driving force responsible for the bleeding, it is wise to consider decompressing the portal venous system using TIPS, assuming that pre- and postsinusoidal causes of portal hypertension have been ruled out. There have been numerous reports on the successful use of TIPS in this setting [7]. TIPS may offer the best chance to prevent future rebleeding when compared to more conservative therapies such as suture ligation, sclerotherapy, and intravascular coil or glue embolization [8]. However, the rates of rebleeding may still be as high as 25% [9]. A common reason to forgo performing TIPS, as was the case with the current report, is the presence of decompensated liver failure and hepatic encephalopathy. It is well known that TIPS formation increases the likelihood and severity of hepatic encephalopathy, and it is estimated that 30% of patients who receive TIPS for stomal variceal bleed will develop hepatic encephalopathy [9]. Shunt creation also poses the risk of interfering with future liver transplantation, assuming that the patient is an appropriate candidate. For example, improper placement of TIPS stent may impair a surgeon's ability to connect a new liver to the portal and hepatic veins. Alternatively, there have been reports of using a transjugular transhepatic approach without creation of a permanent shunt [10].

Another described successful approach to treat symptomatic peristomal varices has been through a percutaneous transhepatic route [10–12]. This gains direct access into the portal system through the liver parenchyma without creating portosystemic shunting. Although this approach has been successfully used, it carries a significant risk of bleeding, particularly because of the underlying liver disease and commonly elevated INR [13]. The percutaneous transsplenic approach offers a large target in the splenic vein, especially when engorged from portal hypertension. However the tortuous nature of the splenic vein and the risk of damaging surrounding structures make this approach technically difficult and would likely increase periprocedural bleeding risk. It is likely for these reasons that the transsplenic approach has not been reported upon in this emergency setting. A final, more recently described approach is balloon-occluded retrograde transvenous occlusion (BRTO) [12–14]. This adopts the technique commonly used to treat gastric varices and applies it to the portosystemic shunt at the stoma.

Direct percutaneous puncture of the intra-abdominal varix with subsequent embolization has been reported as safe and effective [15–18]. As was in our case, the direct percutaneous puncture of the varix can be easily performed under ultrasound guidance. Once accessed, the landing zones for subsequent embolic material are almost immediately available, which theoretically decreases the time to hemostasis. Again, it is important to consider the individual patient's candidacy and need for future liver transplant and to assure that intervention will not impede this candidacy. Despite this particular patient's poor candidacy for liver transplantation, it is worth noting that the described direct percutaneous approach has virtually no risk of interfering with future liver transplantation. In our report, coils were used followed by Gelfoam to embolize the portal venous inflow. Coils and Gelfoam were chosen due to availability and operator comfort. Other reports confirm that coils alone or in conjunction with Gelfoam are both safe and effective, although the risk of coil migration must be taken seriously when choosing size and landing zone [16, 17]. Other embolic materials, such as Histoacryl glue and iodized oil, have been used with success as well [15]. At this time, embolic material should be chosen based on operator comfort as there is no consensus on a superior material in this clinical setting. As detailed above, embolization of the feeding vessels does not treat the underlying portal hypertension. Thus, this approach is of limited value in isolation in patients who are good candidates for TIPS procedure. However, when a patient has significant relative contraindications for TIPS, percutaneous embolization via direct varix sac puncture is a safe and feasible option.
